# Bioactive peptides of marine organisms: Roles in the reduction and control of cardiovascular diseases

**DOI:** 10.1002/fsn3.4183

**Published:** 2024-04-23

**Authors:** Jia Du, Miao Xiao, Naomi Sudo, Qinghua Liu

**Affiliations:** ^1^ College of Materials and Environmental Engineering Hangzhou Dianzi University Hangzhou China; ^2^ Suzhou Health‐originated Bio‐technology, Ltd. Suzhou China; ^3^ Wisdom Lake Academy of Pharmacy Xi'an Jiaotong‐Liverpool University Suzhou China

**Keywords:** anticoagulant activity, antihypertensive activity, bioactive peptides, cardiovascular diseases, functional food, marine organisms

## Abstract

Cardiovascular diseases (CVDs) affect the quality of life or are fatal in the worst cases, resulting in a significant economic and social burden. Therefore, there is an urgent need to invent functional products or drugs for improving patient health and alleviating and controlling these diseases. Marine bioactive peptides reduce and control CVDs. Many of the predisposing factors triggering CVDs can be alleviated by consuming functional foods containing marine biopeptides. Therefore, improving CVD incidence through the use of effective biopeptide foods from marine sources has attracted increasing interest and attention. This review reports information on bioactive peptides derived from various marine organisms, focusing on the process of the separation, purification, and identification of biological peptides, biological characteristics, and functional food for promoting cardiovascular health. Increasing evidence shows that the bioactivity and safety of marine peptides significantly impact their storage, purification, and processing. It is feasible to develop further strategies involving functional foods to treat CVDs through effective safety testing methods. Future work should focus on producing high‐quality marine peptides and applying them in the food and drug industry.

## Graphical abstract

This review reports information on bioactive peptides derived from various marine organisms, focusing on the process of preparation, isolation, and purification of biological peptides, biological characteristics, and functional food in promoting cardiovascular health. Increasing evidence shows that the bioactivity and security of marine peptides have significant impact on storing, purification, and processing. It is feasible to develop further strategy of functional foods to treat cardiovascular (CVD) diseases through perfect safety testing methods.

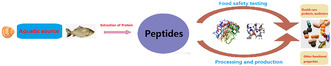

## 1 INTRODUCTION

Cardiovascular diseases (CVDs) are systemic vascular diseases in the heart and peripheral blood vessels, including coronary heart diseases, cerebrovascular diseases, peripheral arterial diseases, rheumatic heart diseases, congenital heart diseases, deep vein thrombosis, and pulmonary embolism (World Health Organization [WHO], 2015). CVDs are often caused by a variety of factors, including disease factors (dyslipidemia, diabetes, etc.), genetic factors (preexcitation syndrome, hypertrophic cardiomyopathy, etc.), and poor lifestyle habits (long‐term high‐sodium, low‐potassium diet, etc.)(Jamee Shahwan et al., 2019; Woteki & Thomas, 1992). CVDs are severe threats to human health, especially in middle‐aged and old individuals, and their mortality and morbidity rates are very high. Even if modern medicine has advanced and perfect treatment, many patients are still at risk of death. According to the WHO, CVDs were severe chronic diseases, and there were 17.5 million deaths from CVDs in 2012 worldwide (Lopez & Murray, 1998; WHO, 2017). Survey data show that the number of people who die from CVDs will reach 23.6 million people in 2030 (WHO, 2015). The rapid increase in the burden of CVDs in countries around the world is caused by dramatic demographic changes and by the impact of the globalized economy. Therefore, the invention and production of cardiovascular drugs and health products have become hot topics.

Bioactive peptides are multifunctional compounds derived from proteins that play critical physiological roles in organisms (Wang et al., 2024). They are isolated from various organisms, including animals, plants, and microorganisms, and obtained through artificial chemical synthesis or bioengineering methods. These proteins have physiological functions in cell metabolism, physiological regulation, and the transmission of biological information (Chi & Wang, 2023). Bioactive peptides range from complex long‐chain or ring polypeptides to small dipeptides with only two amino acids. The molecular structure of bioactive peptides lies between amino acids and proteins and is a protein fragment. They are complex and diverse in structure and are derived by glycosidation, phosphorylation, or acylation. Once released from the original protein, they perform a different function (Kim et al., 2013; Mirzaei et al., 2021). Recent research has suggested that the structure, composition, and sequence of amino acids affect the physiological functions of bioactive peptides. According to the Bioactive Peptides Database (2019 BIOPEP UWM), at least 4300 genes have been identified and classified, and they have been widely studied by researchers due to their hormone action, regulation of the immune system, their antioxidant, antihypertensive, antimicrobial, opioid agonistic, immunomodulatory, prebiotic, mineral binding, antithrombotic, and hypocholesterolemic effects (Hartmann & Meisel, 2007; Kitts & Weiler, 2003; Kovacs‐Nolan et al., 2005; Meisel, 1997; Minkiewicz & Darewicz., 2019).

Worldwide, water covers 80% of the earth's water and has abundant bioactive peptide resources. Marine organisms are rich in protein peptides and have good application prospects in the health products industry, pharmaceuticals, and medical beauty. In recent decades, peptides from marine organisms have been extracted from algae, fish, mollusks, crustaceans, and marine byproducts (shellfish, skins, guts, and muscles). Diverse activities for CVDs have been found in marine bioactive peptides, including cardioprotective, antioxidant, antihypertensive, cholesterol‐lowering, bacteriological inhibition, antidiabetic, antiviral, antiobesity, and tumor inhibition effects (Betoret et al., 2011; Kim et al., 2009; Kim & Mendis, 2006; Najafian & Babji, 2012). Marine bioactive peptides are multifunctional and widely used in CVD prevention and treatment practices as health products, diagnostic reagents, drugs, and vaccines (Pfeuffer & Schrezenmeir, 2000).

This review focuses on bioactive peptides reported from marine sources, such as algae, fish, mollusks, crustaceans, and marine byproducts. This review summarizes the research on purification and isolation techniques, biological characteristics, functional characteristics, and functional food applications. Furthermore, bioactive peptides with properties relevant to CVD prevention and treatment have also been analyzed.

## 2 PREPARATION AND IDENTIFICATION OF BIOACTIVE PEPTIDES FROM MARINE SOURCES

Bioactive peptides of marine sources with 3–40 amino acid residues are inactive within the sequences of the parent protein but are active when released from organisms through gastrointestinal digestion and food processing, including enzymatic hydrolysis, fermentation, acid–base extraction, hot water extraction, or a combination of different techniques (Ge et al., 2023). Bioactive peptides of marine organisms are usually made by in vitro enzymatic hydrolysis of various marine resources. Current enzymatic hydrolysis methods generally include a single enzymatic hydrolysis method and two or more protease complex enzymatic hydrolysis methods. Proteolytic enzymes from algae, mollusks, fish, crustaceans, and other marine organisms act in the enzymatic hydrolysis of samples to produce protein peptides for use in medicines, health products, cosmetics, and other industries. However, enzymatic hydrolysis is related to the selection of protease, hydrolysis time and temperature, and different conditions have different effects on the degree of hydrolysis, thus affecting the type of peptide generated (Korhonen & Pihlanto, 2006). In addition, molecular weight is another factor that affects the functional properties of bioactive peptides (Qian et al., 2007). The preparation methods and peptide activities of the marine origin used for CVD treatment and prevention are shown in Table 1.

**TABLE 1:** Preparation methods and peptide activities derived from marine origin.

Marine organism	Preparation method	Biological effect	Biological characteristics	Reference
Porphyra dioica	Alkaline protease and flavor protease hydrolysis	Angiotensin‐I‐converting enzyme (ACE) inhibitory activity and dipeptidyl peptidase IV (DPP‐IV) inhibitory activity	Antioxidant and antihypertensive	Cermeno et al. (2019)
Microalgae	Gastrointestinal digestive hydrolysis	ACE inhibitory activity	Antihypertensive	Chen et al. (2020)
New Zealand green‐lipped mussel (*Perna canaliculus*)	Pepsin hydrolysis	ACE inhibitory activity	Antioxidant and antihypertensive	Jayaprakash and Perera (2020)
Common carp (*Cyprinus carpio*)	Papain hydrolysis	DPP‐IV inhibition	Antidiabetes and antihypertension	Zhang et al. (2021)
*Hippocampus abdominalis*	Alkaline protease hydrolysis	ACE inhibitory activity and alkyl radical‐scavenging activity	Antioxidant and antihypertensive	Kim et al. (2019)
Silver carp (*Hypophthalmichthys molitrix*)	Neutral proteolytic hydrolysis	DPP‐IV and ACE inhibition	Antihypertensive	Zhang, Cao, et al. (2019), Zhang, Gao, et al. (2019), Zhang, Liu, et al. (2019)
Rainbow trout (*Oncorhynchus mykiss*)	Alkaline protease hydrolysis	DPP‐IV and ACE inhibition	Antidiabetes and antihypertension	Ketnawa et al. (2018)
Marine fish cobia (*Rachycentron canadum*)	Complex protease hydrolysis	ACE inhibitory	Antihypertensive	Lin et al. (2019)
Dried bonito (*Katsuobushi*)	Complex protease hydrolysis	Angiotensin I‐converting enzyme inhibitor	Antihypertensive	Kouno et al. (2005)

Marine biological polypeptides extracted by protein degradation methods contain many impurities. Therefore, further separation and purification technology is needed to improve the physical activity and purity of polypeptides. The purification of active peptides is necessary for the production of consumer products. Currently, the main extraction methods of marine peptides are ultrafiltration, gel filtration chromatography, ion‐exchange chromatography, and reversed‐phase high‐performance liquid chromatography (RP‐HPLC; Hu et al., 2023; Ishak & Sarbon, 2017; Park et al., 2022). For example, the immunoregulatory peptides from *Stolephorus chinensis* were prepared via process optimization, ultrafiltration, ion‐exchange chromatography, and RP‐HPLC (Xu et al., 2020).

Ultrafiltration technology is a nanoscale film separation technology that uses the pressure difference on both sides of the film as the driving force. The solution can achieve separation, purification, and concentration when passing through the filter membrane aperture. The ultrafiltration method is simple and widely used in the study of peptides in marine products (Santiaguín‐Padilla et al., 2022; Vicente et al., 2022; Wang et al., 2022; Zhong et al., 2021). Ultrafiltration technology has a stable effect, low energy consumption, high efficiency, and no pollution, but this technology is only suitable for the initial separation of peptides and cannot separate a specific peptide (Sridhar et al., 2021).

Gel filtration chromatography is also known as molecular exclusion chromatography. The main principle is to use gel action to separate the substances according to different molecular sizes and to achieve separation and purification. The gel used in this method is an inert carrier with no charge and good separation. This method does not require organic solvents in the separation process, so the product is not easy to denature. However, this method generally needs to be combined with other separation and purification methods to obtain higher‐activity and higher‐purity polypeptides (Chen et al., 2021).

Ion‐exchange chromatography is also widely used for the extraction of marine products and their byproducts. For example, Teixeira and Mendes (2022) used an ion‐exchange chromatography method to accurately quantify phosphates in hake fillets. RP‐HPLC is the standard method for purifying polypeptides from marine products (Cian et al., 2021). The main principle is to use the polarity of the mobile phase to decrease the binding effect of the nonpolar fixed phase and the nonpolar polypeptide molecules so that the solute molecules are purified (Toll et al., 2005). Hu et al. (2019) used consecutive chromatography and electrospray ionization‐mass spectrometry (ESI MS) to purify and identify antioxidants from round scad (*Decapterus maruadsi*) hydrolysates.

Mass spectrometry (MS), electrospray ionization MS (ESI MS), matrix‐assisted laser desorption ionization‐time‐of‐flight MS (MALDI‐TOF MS), liquid chromatography–mass spectrometry (LC–MS), and hydrophilic interaction liquid chromatography (HILIC), as well as combinations of various instruments, are commonly used to identify isolated and purified bioactive peptides from marine products and their byproducts (Zaky et al., 2021). Suitable proteolytic enzymes and efficient extraction techniques were used to develop high‐quality biopeptides and apply them in the business field.

## 3 BIOLOGICAL CHARACTERISTICS OF BIOACTIVE PEPTIDES FROM MARINE SOURCES IN CVD TREATMENT

The occurrence and development of CVDs are correlated with the interaction of many risk factors. For example, dyslipidemia and hypertension induce atherosclerosis and thrombosis, which activate vascular inflammation, oxidative stress, hypercoagulability, and the sympathetic and renin–angiotensin systems (Siti et al., 2015; Sowers et al., 2001). These cascading behaviors aggravate CVDs. Bioactive peptides from marine sources have antiatherosclerotic, antihypertensive, antioxidant, antihyperlipidemic, and anticoagulant activities, and their functions have been extensively studied for the treatment of CVDs.

### 3.1 Antiatherosclerotic peptides

Atherosclerosis refers to the deposition of lipids and other blood components in the intima of the artery, the proliferation of smooth muscle cells, and an increase in collagen fibers, resulting in the formation of necrotic lipid‐containing cone‐like lesions and hardening of the vascular wall. Atherosclerosis can lead to myocardial infarction, ischemic cardiomyopathy, stroke, and peripheral artery disease and is the main cause of CVD (Wolf & Ley, 2019). Atherosclerosis is the leading cause of death worldwide, accounting for 84.5% of CVDs. Studies have shown that some algae‐derived polypeptides have antiatherosclerotic activity and are widely used in food and pharmaceutical products. For example, Vo and Kim (2013) reported that two bioactive peptides (P1 (LDAVNR; 686 Da) and P2 (MMLDF; 655 Da)) isolated from the gastric enzymatic hydrolysate of *Spirulina maxima* were effective in preventing early atherosclerosis. *Chlorella* 11‐peptide exhibited practical anti‐inflammatory effects, and these inflammatory indices are important markers of atherosclerosis. Shih et al. (2013) demonstrated that *Chlorella* 11‐peptide is a promising biomolecule for preventing chronic inflammatory‐related vascular diseases. Platelet‐activating factor acetylhydrolase (PAF‐AH) inhibitory peptides isolated from the red macroalga *Palmaria palmata* have good effects in preventing atherosclerosis (Fitzgerald et al., 2013). Pei et al. (2022) isolated a novel peptide from microalgae *Isochrysis zhanjiangensis*. It exhibits anti‐apoptosis and anti‐inflammation in oxidized low‐density lipoprotein (ox‐LDL) and induces human umbilical vein endothelial cells (HUVECs) to improve atherosclerosis. Several microalgal peptides have antiatherosclerotic activity.

The mechanisms underlying the antiarteriosclerosis effects of these agents include reductions in the levels of cell molecules, the activation of red blood cell (RBC) factors, the inhibition of acetylhydrolase, and the treatment of atherosclerotic cells with the histamine factor, which is an anti‐inflammatory reactive oxygen species. There is insufficient theoretical research on the use of microalgal peptides for the treatment and prevention of arteriosclerosis. As a potentially crucial raw material for the treatment of CVDs, microalgal peptides with good water solubility should be further developed and applied in pharmaceutical products.

### 3.2 Antihypertensive peptides

Hypertension is increasingly prevalent worldwide and is the main risk factor for CVD (Kannel & Higgins, 1990). Angiotensin I‐converting enzyme (ACE) plays key roles in the regulation of blood pressure by promoting the conversion of angiotensin‐I to angiotensin‐II. Therefore, inhibiting the conversion process of angiotensin is the key target for preventing elevated blood pressure. Food proteins of marine organisms are important sources of ACE‐inhibitory (ACEi) peptides, which have potential antihypertensive activity. ACEi peptides derived from various fish species (e.g., boarfish, tuna, *Rachycentron canadum*, bonito, monkfish, and salmon) have good antihypertensive effects (Hayes et al., 2016; Itou & Akahane, 2004; Lee et al., 2010; Neves et al., 2017). For instance, Hu et al. (2023) isolated and characterized collagen and ACEi peptides from the swim bladders of monkfish (*Lophius litulon*). Seafood byproduct proteins are potential ACEi peptide sources, and the prepared ACEi peptides from skipjack tuna dark muscle are beneficial functional food components for treating hypertension and CVD (Qiao et al., 2022; Zheng et al., 2022). Zhu et al. (2022) reported that ACEi peptides derived from miiuy croaker swim bladders are health‐promoting functional products that can be used as supplementary treatments for hypertension and CVD. Suo, Zheng, et al. (2022) derived novel ACEi peptides from the tuna byproduct milts. Chan et al. (2022) reported that bioactive peptides isolated from the heads and bones of hybrid groupers could be used as functional foods/ingredients with potential ACE inhibitory and antioxidant effects. Lin et al. (2019) prepared and identified antihypertensive peptides isolated from in vitro gastrointestinal digestion of marine cobia skin hydrolysates. Matsumura et al. (1993) isolated ACEi peptides derived from bonito bowels.

Among the marine living resources, turtle eggs, mussels, and algae also have the potential to produce ACEi peptides. Rawendra et al. (2013) derived a novel ACEi peptide from the proteolytic digestion of Chinese soft‐shelled turtle egg white proteins. Pujiastuti et al. (2017) derived ACEi peptides from soft‐shelled turtle yolk using two orthogonal bioassay‐guided fractionations. Suo, Zhao, et al. (2022) isolated 17 ACEi peptides from the protein hydrolysate of the blue mussel (*Mytilus edulis*). These ACEi peptides could be used as natural ingredients for the development of products with antihypertensive functions. Neves et al. (2016) reported that mussel meat protein hydrolysates have potential as functional food ingredients for managing diseases such as type II diabetes and hypertension. You et al. (2023) reported that mussel‐derived ACEi peptides improve spontaneous hypertension in rats. The protein hydrolysates of *Mytilus edulis* and *Crassostrea gigas* also appear to be the source of ACEi peptides (Je, Park, Byun, et al., 2005; Je, Park, Jung, et al., 2005).

Antihypertensive peptides obtained from *Undaria pinnatifida*, Microalgae *Palmaria palmata*, the microalga *Chlorella vulgaris*, *Ulva intestinalis*, and *Gracilariopsis lemaneiformis* all showed in vitro ACE inhibitory activity (Deng et al., 2018; Fitzgerald et al., 2012; Sheih, Fang, et al., 2009; Sheih, Wu, et al., 2009; Suetsuna & Nakano, 2000; Sun et al., 2019). For example, the peptide fractions that inhibited ACE were separated from the peptic digests of two microalgae, *Chlorella vulgaris* and *Spirulina platensis*, by ion‐exchange chromatography and gel filtration, which has marked antihypertensive effects on hypertensive rats (Suetsuna & Chen, 2001). Suetsuna et al. (2004) reported that the peptide *Undaria pinnatifida* (wakame) has an antihypertensive impact on spontaneously hypertensive rats. Sun et al. (2019) successfully prepared and identified ACEi peptides from the marine Macroalga *Ulva intestinalis*. Thus, marine organisms are essential resources of antihypertensive peptides.

### 3.3 Antioxidant peptides

Oxidative stress, the constant production of reactive oxygen species (ROS) combined with the activation of the oxidative defense system, is a crucial cause of CVDs. ROS are free radicals with oxygen, including hydroxyl radicals (·OH), superoxide radicals (O_2_·^−^), hydrogen peroxide (H_2_O_2_), oxygen (O_2_), hypochlorite (ClO^−^), and nitric oxide radicals (NO; Cai et al., 2022). Under normal circumstances, these substances are quickly removed from the body. Nevertheless, if the body produces too much or cannot be removed quickly, pathological damage, biological macromolecule damage, and dysfunction of human tissue cells can occur, which eventually leads to a variety of diseases, including CVD, nephropathy, neurological disease, cancer, etc. (Chi et al., 2015; Lobo et al., 2010; Zhang, Cao, et al., 2019; Zhang, Gao, et al., 2019; Zhang, Liu, et al., 2019). Therefore, antioxidants are vital for neutralizing excess free radicals or preventing the formation or decay of free radicals. Antioxidant peptides have the potential to improve CVD risk. The relationship between antioxidant peptides and CVDs has been confirmed by extensive research and has received increased amounts of attention in the last 10 years.

Extractable antioxidant peptides from nature have been studied in different organisms. Antioxidant peptides, mainly with a low molecular weight (MW) of 3 kDa, are extracted from marine organisms. Most antioxidant peptides produced by enzymatic hydrolysis are obtained from marine organisms, including fish, mollusks, echinoderms, and algae (Admassu et al., 2018; Hu et al., 2023). Antioxidant peptides isolated from water resources have antioxidative and antihypertensive effects, both of which are related to the treatment of CVDs. The ability of antioxidative peptides to scavenge different kinds of free radicals, including hydroxyl radicals (OH·), hydrogen peroxide (H_2_O_2_), 1,1‐diphenyl‐2‐picrylhydrazyl (DPPH) radical, and 2,2‐azino‐bis(3‐ethylbenzthiazoline)‐6‐sulfonic acid (ABTS) radical, is investigated. Some research have also shown that antioxidant peptides can protect deoxyribonucleic acid (DNA) and the absorbance capacity of superoxide radicals (O_2_·^−;^ Heo & Jeon, 2008; Sheih, Fang, et al., 2009; Sheih, Wu, et al., 2009). The antioxidant peptides extracted from *Chlorella vulgaris* and *Ishige okamurae* had a protective effect on DNA. Najafian and Babji (2018) reported that the presence of hydrophobic amino acids (Ile and Leu) and the presence of acidic (Asp) and primary (His) amino acids in the peptide sequences were the reasons for the high antioxidant activity of the fermented anchovy fish (*Budu*). The mechanisms of action of antioxidant peptides are not fully understood. Overall, the current study revealed that the antioxidant action of these peptides is attributed to the cooperative effects of metal ion chelation, free radical scavenging, and singlet oxygen quenching (Kitts & Weiler, 2003).

Among these marine organisms, algae have a considerable ability to scavenge free radicals. For example, the antioxidative peptides derived from *Chlorella vulgaris* can effectively destroy many kinds of free radicals and prevent cellular damage (Sheih, Fang, et al., 2009; Sheih, Wu, et al., 2009). Antioxidant peptides derived from *Gracilariopsis lemaneiformis* protein hydrolysates had a protective effect on H_2_O_2_‐induced cell oxidative damage (Hu et al., 2023). Research has shown that antioxidant peptides extracted from fish and mollusks have potential applications as functional foods and drugs. For example, antioxidant peptides from the protein hydrolysate of skipjack tuna milt can be natural antioxidant ingredients used in pharmaceutical and functional products (Wang et al., 2022). Korczek et al. (2018) reported that bioactive peptide hydrolysates in fish products could represent an excellent substitute for synthetic drugs to treat hypertension diseases and CVDs. Antioxidant peptides derived from *Piaractus brachypomus* fish meat exhibited significantly greater antioxidant activity and could be biofunctional ingredients in food and nutraceutical applications (Hashem et al., 2023). Xia et al. (2017) reported that peptides derived from pearl oyster (*Pinctada martensii*) mantle type V exhibit more potent antioxidant activity than tilapia (*Oreochromis niloticus*) scale type I collagen, and *P. martensii* is a good source of natural antioxidants in the food‐processing industry. Zhang et al. (2020) reported that antioxidant peptides purified from  *Mytilus coruscus* improved cell viability and ameliorated morphological damage in human umbilical vein endothelial cell (HUVEC). Park et al. (2020) purified a novel NCWPFQGVPLGFQAPP peptide from clam worms (*Marphysa sanguinea*). This NCW peptide with antioxidant and anti‐inflammatory effects could be a good therapeutic agent against inflammation‐related diseases. Ma et al. (2021) purified antioxidant peptides from *Pinctada fucata* with hydroxyl, superoxide radical‐scavenging, and cellular antioxidant activity. Joshi and Abdul (2021) isolated the ACEi peptide derived from a *Squilla species* with ACE inhibition and antioxidant potential. Antioxidant peptides isolated from monkfish muscle could serve as powerful antioxidants for the treatment of some liver diseases and health care products associated with oxidative stress (Hu et al., 2020). Oh et al. (2021) reported that seahorse‐derived peptides might be promising agents for oxidative stress‐related CVDs. Safari and Yaghoubzadeh (2020) suggested that antioxidant peptides extracted from sea cucumber (*Holothuria leucospilota*) could be used as a natural source of antioxidant compounds in the pharmaceutical and food industries. Antioxidant peptides isolated from Antarctic krill (*Euphausia superba*) showed strong reducing power, protective ability against H_2_O_2_‐damaged plasmid DNA, and lipid peroxidation inhibition ability (Zhang et al., 2021).

As mentioned above, oxidative stress is an important pathogenic factor of CVD because it is involved in the occurrence of CVD. The use of antioxidant peptides is an important breakthrough in the treatment of CVDs. Antioxidant peptides from marine sources are abundant and could be an essential alternative to expensive drug‐derived compounds.

### 3.4 Antihyperlipidemic peptides

Hyperlipidemia is another critical risk factor for the development of CVDs. Hyperlipidemia is positively associated with the likelihood of developing CVDs. Hypercholesterolemia and hypertriglyceridemia are essential diseases caused by hyperlipidemia and are common in patients with CVD. Bioactive peptides derived from fish protein hydrolysates, algal fucans, galactans, and alginates have hypocholesterolemic properties, and these properties change the plasma profile from atherogenic to cardioprotective. Various studies have reported that marine peptides are effective at preventing and treating hypercholesterolemia and hypertriglyceridemia. For example, C‐phycocyanin protein isolated from *Spirulina platensis* reduced the levels of total cholesterol, high‐density lipoprotein, and triacylglycerols in rats and rabbits (Colla et al., 2008; Nagaoka et al., 2005). Bioactive peptides extracted from microalgal glucans (*polysaccharides*) activate the immune system and exert antioxidant and hypocholesterolemic effects (Villarruel‐López et al., 2017). The protein hydrolysate of fish reduced plasma total cholesterol, increased the proportion of high‐density lipoprotein‐cholesterol, and reduced acyl‐coenzyme A (CoA): cholesterol acyltransferase activity in the liver of Zucker rats (Wergedahl et al., 2004). Wang et al. (2023) reported that novel hypocholesterolemic peptides derived from silver carp muscle, and that these peptides possessed dual hypocholesterolemic functions, including inhibition of cholesterol absorption and promotion of peripheric low‐density lipoprotein (LDL) uptake. Fish protein hydrolysates from sardine, horse mackerel, axillary seabream, bogue, small‐spotted catshark, and blue whiting are suitable formula ingredients for cholesterol‐lowering supplements.

The number of studies on the antihyperlipidemic effects of marine biopeptides is very limited, so the mechanism by which protein peptides reduce blood lipids has received increasing attention. To date, the mechanisms underlying the antihyperlipidemia effects of bioactive peptides are not fully clear. Some research has shown that peptide structure influences plasma cholesterol levels, and low ratios of methionine–glycine and lysine–arginine are good for hypocholesterolemic effects (Karami & Akbari‐adergani, 2019). Shibata et al. (2007) showed that *Chlorella* enhanced hepatic cholesterol catabolism by upregulating cholesterol 7 alpha‐hydroxylase in rats. Some researchers have suggested that the hypocholesterolemic effects of biopeptides are due to their specific components. For example, Villarruel‐López et al. (2017) found that microalgal peptides activated the immune system, exerted antioxidant and hypocholesterolemic effects, and inhibited specific receptors involved in CVD. These biological activities mainly depend on their particular chemical constituents. In short, marine bioactive peptides, which have functional characteristics, have been made into health care products to decrease hyperlipidemia.

### 3.5 Anticoagulant peptides

Thrombosis is the formation of blood clots in an artery or vein and is the main cause of three major fatal CVDs (heart disease, stroke, and venous thromboembolism). Blood coagulation factors can stop bleeding and repair damaged blood vessels. Anticoagulants are used as hemostatic drugs to reduce platelet aggregation. At present, the main commercial anticoagulants are heparin, low‐molecular‐weight heparin, warfarin, aspirin, and clopidogrel. Serious complications, thrombocytopenia development, and immune response elicitation limit their long‐term application. Clopidogrel and aspirin are antiplatelet drugs that are indispensable for primary or secondary prevention of ischemic cardio‐cerebrovascular disease but have several side effects, including increased risk of bleeding, drug resistance, gastrointestinal toxicity, and increased platelet reactivity.

Some peptides extracted from marine organisms with anticoagulant properties have received attention in recent years because they are noncytotoxic and excellent alternative resources. Polysaccharides, proteoglycans, and proteins are the main macromolecules derived from marine anticoagulant compounds (Church et al., 1989; Rajapakse et al., 2005). Marine active peptides with anticoagulant activity usually have low molecular weights, which can reduce allergic reactions caused by intestinal system absorption (Kong et al., 2014). The main sources of anticoagulant peptides from marine sources, including algae, starfish, mussels, and marine echiuroid worms, have been reported to be spoonworm (Cheng et al., 2018; Indumathi & Mehta, 2016; Jo et al., 2008; Jung & Kim, 2009; Koyama et al., 1998; Yasuda et al., 2004). For example, a potent and novel anticoagulant peptide was enzymatically isolated from the edible seaweed *Porphyra yezoensis* (commercially known as Nori; Indumathi & Mehta, 2016). An anticoagulant peptide was isolated, purified, and identified from the pepsin hydrolysate of oyster (*Crassostrea gigas*), which could potently prolong the activated partial thromboplastin time and thrombin time (Cheng et al., 2018). Jung and Kim (2009) isolated an anticoagulant oligopeptide from blue mussel (*Mytilus edulis*) that effectively prolonged coagulation time. An anticoagulant peptide from a marine echiuroid worm (*Urechis unicinctus*) has been made into new health care products or pharmaceutical materials (Jo et al., 2008).

**FIGURE 1:**
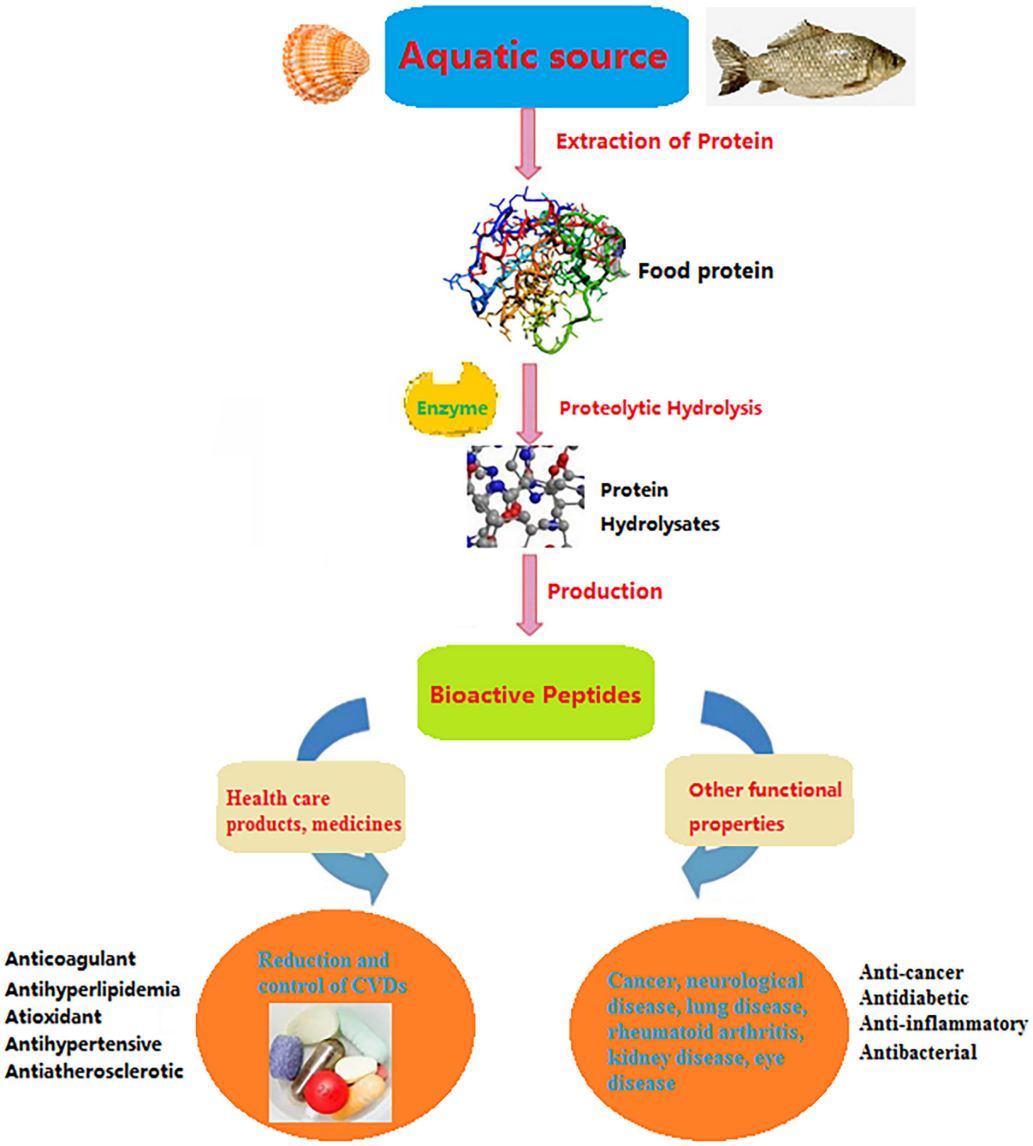
Extraction, processing and application of bioactive peptides from marine proteins.

Currently, most of the anticoagulants used on the market are polysaccharide components, but the trigger of anticoagulant function may be a mixture of polysaccharides, proteins, or peptides (Koyama et al., 1998). The molecular mechanism underlying the relationship between the antihemagglutination factor and coagulation function is insufficient and has not been fully characterized. The use of anticoagulant proteins or peptides from marine organisms is limited, which leads to insufficient information on the anticoagulant activity of proteins. Thus, future studies should focus on anticoagulant bioactive peptides from marine sources. The isolated marine peptides and their functions in the treatment of CVDs are shown in Table 2. The extraction, processing, and application of the marine peptides are shown in Figure 1.

**TABLE 2:** Biological characteristics of the bioactive peptides derived from marine organisms.

Activity	Species	Effect	Reference
Antiatherosclerotic	*Spirulina maxima*	Suppress histamine‐induced endothelial cell activation	Vo and Kim (2013)
	*Chlorella*	Prevent chronic inflammatory‐related vascular diseases	Shih et al. (2013)
	*Palmaria palmata*	Prevent atherosclerosis	Fitzgerald et al. (2013)
	*Isochrysis*	Exhibit anti‐apoptosis and anti‐inflammation	Kishimoto et al. (2016)
Antihypertensive	Marine cobia skin	Inhibit ACE	Lin et al. (2019)
	Bonito bowels	Inhibit ACE	Matsumura et al. (1993)
	Chinese soft‐shelled turtle egg	Inhibit ACE	Rawendra et al. (2013)
	Soft‐shelled turtle yolk	Inhibit ACE	Pujiastuti et al. (2017)
	Mussel meat	Cure type II diabetes and hypertension	Neves et al. (2016)
	*Mytilus edulis*	Inhibit ACE	Je, Park, Byun, et al. (2005)
	*Crassostrea gigas*	Inhibit ACE	Je, Park, Jung, et al. (2005)
	*Ulva intestinalis*	Inhibit ACE	Sun et al. (2019)
	*Mytilus edulis*	Antihypertensive effects in vivo	You et al. (2023), Suo, Zhao, et al. (2022)
	Skipjack tuna muscle	Beneficial components for functional food against hypertension and cardiovascular diseases	Zheng et al. (2022)
	Miiuy croaker swim bladders	With the highest ACEi activity	Zhu et al. (2022)
	Skipjack tuna muscle and milts	Antihypertensive effects	Qiao et al. (2022), Suo, Zheng, et al. (2022)
	Monkfish swim bladders	Antihypertensive effects	Hu et al. (2023)
	Heads and bones of hybrid groupers	With potential ACE inhibitory and antioxidant effects	Chan et al. (2022)
Antioxidant	Anchovy fish	High antioxidant activity	Najafian and Babji (2018)
	*Chlorella vulgaris*	Prevent cellular damage	Sheih, Fang, et al. (2009), Sheih, Wu, et al. (2009)
	*Pinctada martensii*	Exhibit stronger antioxidant activity	Xia et al. (2017)
	Clam worms	With antioxidant and anti‐inflammatory effects	Park et al. (2020)
	*Pinctada fucata*	With hydroxyl, superoxide radical‐scavenging, and cellular antioxidant activity	Ma et al. (2021)
	Squilla	With ACE inhibition along with antioxidant potential	Joshi and Abdul (2021)
	Monkfish	Serve as powerful antioxidants applied in the treatment of some liver diseases	Hu et al. (2020)
	Seahorse	Ameliorated oxidative stress‐mediated human umbilical vein endothelial cells (HUVECs) injury	Oh et al. (2021)
	Sea cucumber	Antioxidant activity	Safari and Yaghoubzadeh (2020)
	Antarctic krill	Strong reducing power, protective capability against H_2_O_2_‐damaged plasmid DNA, and lipid peroxidation inhibition ability.	Zhang et al. (2021)
	*Mytilus coruscus*	Improved cell viability and ameliorated the morphological damage	Zhang et al. (2020)
	Red‐bellied pacu fish	Antioxidant activities	Hashem et al. (2023)
	Skipjack tuna	Presented significant protective function on H_2_O_2_ oxidative damaged human umbilical vein endothelial cells by increasing the human umbilical vein endothelial cells’ viability and antioxidant enzyme activity	Wang et al. (2022)
	*Gracilariopsis lemaneiformis*	Protective effect exerted by the antioxidant peptide on H_2_O_2_‐induced oxidative damage	Hu et al. (2023)
Antihyperlipidemia	*Spirulina platensis*	Reduce the levels of total cholesterol, high‐density lipoprotein, and triacylglycerols	Nagaoka et al. (2005), Colla et al. (2008)
	Polysaccharides	Activate the immune system or exert antioxidant and hypocholesterolemic effects	Villarruel‐López et al. (2017)
	Silver carp	Inhibit cholesterol absorption and promote peripheric LDL uptake	Wang et al. (2023)
	*Chlorella*	Enhance hepatic cholesterol catabolism by upregulation of cholesterol 7 alpha‐hydroxylase	Shibata et al. (2007)
	Microalgae	Activate the immune system or exert antioxidant activity	Villarruel‐López et al. (2017)
Anticoagulant	*Porphyra yezoensis*	Exhibit higher anticoagulant activity	Indumathi and Mehta (2016)
	*Crassostrea gigas*	Inhibit activity against α‐thrombin	Cheng et al. (2018)
	*Mytilus edulis*	Prolong blood clotting by inhibiting activation of Factor X (FX)	Jung and Kim (2009)
	*Urechis unicinctus*	Inhibit an endogenous blood coagulation factor in the intrinsic pathway	Jo et al. (2008)

## 4 FUNCTIONAL FOOD OF MARINE ORIGIN PROMOTES CARDIOVASCULAR HEALTH

**FIGURE 2:**
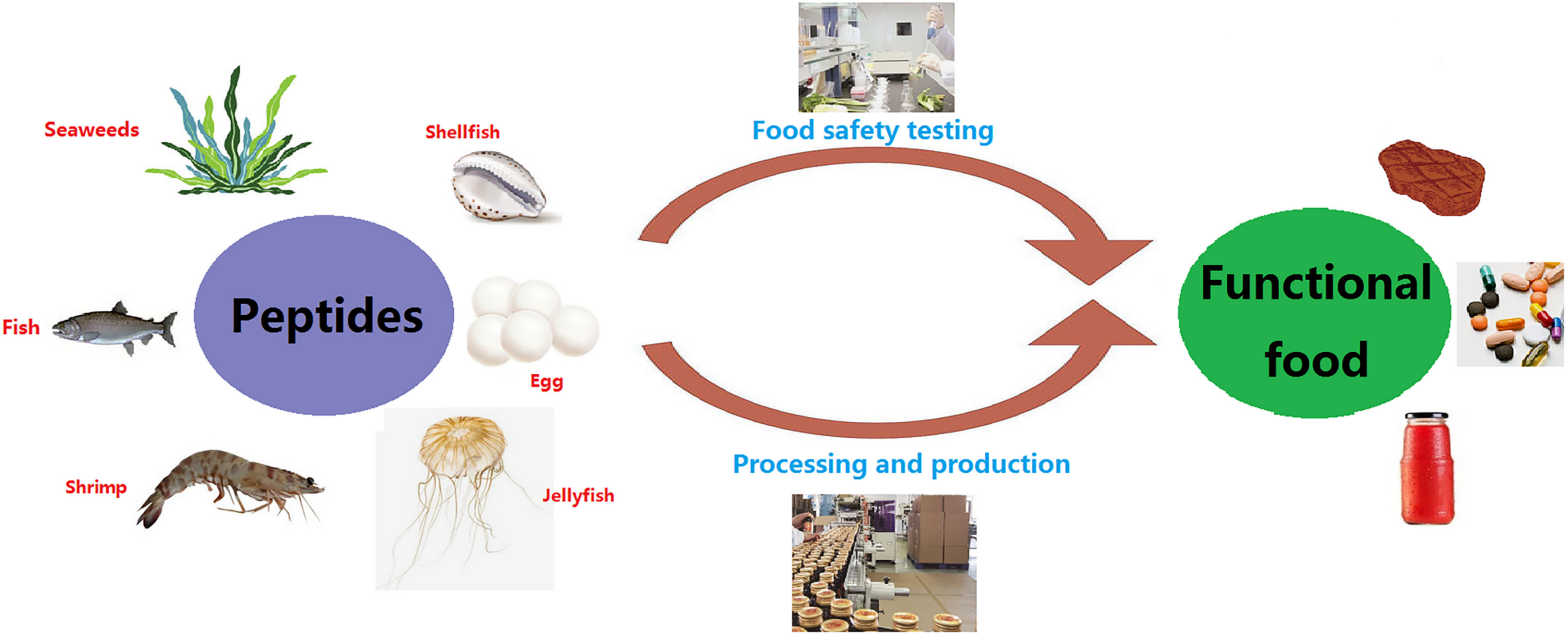
Process for the production and security detection of peptides.

Bioactive peptides from marine resources have gained acceptance from the scientific community as therapeutic drugs and functional food formulations (as shown in Figure 2). As a source of macronutrients, micronutrients, and bioactive components, marine organisms have outstanding application prospects in the functional food and nutritional health industry and contribute to the reduction of risk factors associated with CVDs. Soluble dietary fibers, peptides, phlorotannins, lipids, and minerals are the major compounds isolated from marine organisms and have become the primary raw materials of functional foods. Functional food, a mix of nutrients and pharmaceuticals, is beneficial for reducing the risk of specific diseases or health concerns. Increasing evidence has shown that marine peptides with angiotensin‐converting enzyme inhibitory, antihypertensive, antioxidative, and antidiabetic activities have been successfully processed into functional food.

Studies on the cardiovascular health of seaweeds in meat‐, bread‐, or drink‐based functional foods have increased in recent years. The main objectives of these products are reducing the contents of cholesterol, fat, salt, and functional components and increasing the fatty acid composition. Ultimately, they do not affect the taste or quality of meat products (Cofrades et al., 2012, 2013). In addition, some researchers have evaluated the effect of functional foods on the cardiovascular system, including lipid profile indices, antioxidant enzymes, and arylesterase (Lim & Kim, 2013; Schultz Moreira et al., 2011; Schultz Moreira et al., 2013; Schultz Moreira et al., 2014). For example, Schultz Moreira et al. (2011) demonstrated that seaweeds mixed with restructured meat blocked the effect of hypercholesterolemic agents and gave rise to a new balance of antioxidant enzyme expression. They found that nori‐ and sea spaghetti have a good effect on relieving high cholesterol and blood lipids (Schultz Moreira et al., 2013) and increasing the antioxidant capacity within a noncholesterol‐enriched diet while improving the lipoprotein profile within a cholesterol‐enriched diet (Schultz Moreira et al., 2014).

In addition, sea tangle‐added pork and chicken have decreased postprandial plasma glucose concentrations and reduced insulin secretion in adults with borderline hypercholesterolemia (Lim & Kim, 2013). Hall et al. (2012) suggested that *Ascophyllum nodosum*‐enriched bread reduced energy intake and modulated glycemic and cholesterolemic responses in healthy and overweight males. Increasing evidence shows that drinks with sea algae can also prevent cardiovascular disorders. For example, drinks made from seaweed have ACE inhibitory activities (Nagai et al., 2006). A beverage composed of seaweed fusiforme and onion is used to prevent hypertension (Kim, 2008). Nagai and Yukimoto (2003) reported that beverages made from sea algae are candidate health drinks for patients suffering from cancer, CVD, and diabetes due to their antioxidant activities.

Bioactive peptides derived from fish are healthy components of the diet and have attracted the attention of consumers. Bioactive peptides isolated from fish have also been developed as antihypertensive components in functional foods or nutraceuticals. For example, the active peptides of salmo protein hydrolysate could be used as functional foods for controlling hypertension (Ahn et al., 2012). Surimi, made from olive flounder (*Paralichthys olivaceus*), contains novel antihypertensive peptides and is a healthy ingredient (Oh et al., 2020). The purified peptide isolated from shortfin scad waste hydrolysate has potential antihypertensive properties and can be used as a functional food (Ishak et al., 2021).

Fish skin is rich in protein, various nutrient elements, and bioactive compounds, but it has become one of the most wasted byproducts in the fish processing industry. Gu et al. (2011) identified ACE inhibitory peptides from Atlantic salmon (*Salmo solar*L.) skin. Najafian and Babji (2012) reviewed the techniques used to isolate and characterize these compounds in fish skin and their applications in food and nutraceuticals. Himaya et al. (2012) reported that Pacific cod skin could be used as a functional food because it could control ACE activity and oxidative stress. These researchers found that different peptide sequences in fish skin significantly contribute to ACE inhibitory activity. Much attention has been given to isolating peptides from other marine organisms, such as shrimp, jellyfish, and shellfish, which are also beneficial antihypertensive compounds in functional food resources (Feng et al., 2016; Katano et al., 2003; Liu et al., 2013). Sea cucumber (*Holothuria forskali*) is processed into cans to meet the health and nutritional requirements of consumers, which exhibit antioxidant and antihypertensive functions (Garcia et al., 2019).

The safety of peptides in food applications is also a concern because allergenic or toxic peptides are formed during processing, protein pretreatment, and extraction processes. Although a variety of different bioactive peptides have been isolated from marine organisms, most functional foods are not consumed, so the safety of these peptides is unknown. The safety evaluation of purified peptides in functional foods is unpopular. In short, additional products of marine biological peptides will enter the market as health supplements to promote cardiovascular health due to the continuous efforts of the medical and processing industries. However, the mass production of marine peptides, efficient isolation methods, and their digestibility and safety in vivo require further research. In addition, effective medicines should be further developed after the conversion of marine products into functional foods and nutritional products, which will help address nutrition‐related problems, prevent diseases, and ultimately improve consumer life.

## 5 CONCLUSIONS

Marine bioactive peptides play potential roles in the reduction and control of CVDs. The biological characteristics of the bioactive peptides, including their antiatherosclerotic, antihypertensive, and antioxidant activities and their antihyperlipidemic and anticoagulant effects, were reviewed. The functional effects of bioactive peptides on reducing risk factors associated with CVDs, including hypertension, hyperlipidemia, anticoagulant use, and obesity, were also reviewed. Compared with clinical drugs, marine peptides have a similar mechanism of action toward CVD and can potentially replace some clinical drugs. Many functional foods, health care products, and medicines have been developed by incorporating marine peptides as raw materials. The structure and composition of marine peptides affect their biological activity and function. Although researchers have made specific contributions to the therapeutic potential of marine peptides for CVD treatment, more efforts should be made to discover more functionally active peptides.

The primary raw materials of most functional products are peptides from fish, shrimp, jellyfish, shellfish, and algae. In addition, the physiological and therapeutic properties of marine peptides are affected by the process of preparation and hydrolysis. Moreover, further safety analyses or risk assessments of marine polypeptide health products need to be performed. At present, there is no unified assessment standard of safety risk for allergenic or toxic peptides. With the increasing market demand for multifunctional marine polypeptides, suitable proteolytic enzymes and efficient extraction techniques should be used to develop high‐quality biopeptides for application in the food industry. Further studies on the development of storage, biopeptide extraction, processing methods, and perfect food safety evaluation systems are essential.

## AUTHOR CONTRIBUTIONS

**Jia Du:** Writing – review and editing (lead). **Miao Xiao:** Visualization (supporting). **Naomi Sudo:** Funding acquisition (lead). **Qinghua Liu:** Conceptualization (lead).

## CONFLICT OF INTEREST STATEMENT

The authors declare that they do not have any conflict of interest.

## ETHICAL STATEMENT

This study does not involve any human or animal testing.

## INFORMED CONSENT

Written informed consent was obtained from all study participants.

## Data Availability

No data sharing in the current study.
